# Goals-of-Care Consult and Future Costs, Acute Care and Hospice Use in Matched Cohorts of African Americans and Whites

**DOI:** 10.1093/geroni/igaa057.1681

**Published:** 2020-12-16

**Authors:** Lauren Starr, Connie Ulrich, Scott Appel, Paul Junker, Nina O’Connor, Salimah Meghani

**Affiliations:** 1 University of Pennsylvania School of Nursing, Philadelphia, Pennsylvania, United States; 2 University of Pennsylvania Perelman School of Medicine, Philadelphia, Pennsylvania, United States; 3 University of Pennsylvania Health System, Philadelphia, Pennsylvania, United States

## Abstract

African Americans receive less hospice care and more aggressive end-of-life care than Whites. Little is known about how palliative care consultation to discuss goals-of-care (“PCC”) is associated with future acute care utilization and costs, or hospice use, by race. To compare future acute care costs and utilization and discharge to hospice between propensity-matched cohorts of African Americans with and without PCC, and Whites with and without PCC, we conducted a secondary analysis of 35,154 seriously-ill African American and White adults who had PCC at a high-acuity hospital and were discharged 2014-2016. We found no significant difference between African Americans with or without PCC in mean future acute care costs ($11,651 vs. $15,050, P=0.09), 30-day readmissions (P=0.58), future hospital days (P=0.34), future ICU admission (P=0.25), or future ICU days (P=0.30), but found greater discharge to hospice among African Americans with PCC (36.5% vs. 2.4%, P<0.0001). We found significant differences between Whites with PCC vs. without PCC in mean future acute care costs ($8,095 vs. $16,799, P<0.001), 30-day readmissions (10.2% vs. 16.7%, P<0.0001), future days hospitalized (3.7 vs. 6.3 days, P<0.0001), and discharge to hospice (42.7% vs. 3.0%, P<0.0001). Results suggest PCC decreases future acute care costs and utilization in Whites and, directionally but not significantly, in African Americans; and increases discharge to hospice in both races (15-fold in African Americans, 14-fold in Whites). Research is needed to understand how PCC supports end-of-life decision-making and hospice use across races and how systems and policies can enable effective goals-of-care consultations across settings.

